# Osteoradionecrosis – an update for the general dental practitioner

**DOI:** 10.1038/s41415-026-9953-7

**Published:** 2026-09-11

**Authors:** Alice Rigby, Joshua Twigg, Jaymit Patel, Zaid Ali

**Affiliations:** 918026765771161385189 School of Dentistry, University of Leeds, Leeds, United Kingdom; 186495608548365886589 Restorative Dentistry, Leeds Teaching Hospitals Trust, United Kingdom

## Abstract

This review provides an update for the general dental practitioner (GDP) on osteoradionecrosis (ORN) of the jaws, covering aetiopathogenesis, key diagnostic features, disease classification and basic management principles. ORN can occur as a complication of therapeutic radiotherapy, leading to bone necrosis in the jaws that may be detected radiographically or clinically.

We report on a 48-year-old patient who developed ORN secondary to pre-radiotherapy removal of a wisdom tooth. This patient's disease has been treated effectively, and they have now undergone implant-based restorative oral rehabilitation. Clinical photographs and radiographs have been included to highlight the key diagnostic features of this condition.

Risk factors for ORN including therapeutic dose of radiation, patient, tumour and dental factors are explored. Prevention is a key consideration when managing patients with ORN to reduce the risk of dental disease, as infections, loss of teeth or extractions may precipitate the condition. GDPs have a key role in managing these patients as part of a shared-care model with hospital specialties.

## Key points

- Osteoradionecrosis (ORN) can be defined as bony changes that are detected radiographically and/or clinically in patients following radiotherapy treatment.
- A pre-radiotherapy dental assessment is a key part of the treatment pathway to reduce the risk of ORN.
- The general dental practitioner has an important role in ORN prevention and surveillance as part of a shared-care model for these patients.

## What is osteoradionecrosis?

Osteoradionecrosis (ORN) is a recognised complication of head and neck radiotherapy that results in bone necrosis, with potentially severe consequences for the patient.

Previously, definitions for ORN hinged on clinically detectable ‘exposed bone' for a minimum timeframe (typically eight weeks). However, a recent Delphi process, and a growing body of evidence that ORN can be detected radiographically, has resulted in a new definition being developed.^1^^,^^2^^,^^3^ ORN can be defined as bony changes that are detected radiographically and/or clinically. Radiographic features include pathological fractures as well as lytic or sclerotic areas. Clinical features may include exposed bone directly visible or by probing through a fistula. These changes occur in bone that has received therapeutic levels of radiotherapy, which can result in reduced bone vascularity.^1^^,^^4^ A focus on clinical and radiographic detection is made, with biopsies often avoided as they risk worsening the condition.^5^

### Classification

There are >15 classifications proposed for ORN. Many classification systems necessitate multiple appointments to confirm an ORN diagnosis.^4^ Others only include the mandible^6^^,^^7^ or only evaluate whether ORN is actively progressing.^8^ Commonly used classification systems include Marx, based on response to treatment^9^ and Notani, based on extent of bony involvement.^7^

The recent American Society of Clinical Oncology (ASCO) guidelines suggest use of the newly developed ClinRad classification which received support in expert clinical consensus studies.^1^^,^^4^^,^^5^ The ClinRad classification includes five stages (Table 1).

**Table 1:** ClinRad classification of ORN as described by Watson *et al.*^4^

**Stage**	**Presentation**
Minor bony spicule	Not yet ORN but more common in post-radiotherapy cases e.g., superficial sequestrae
Stage 0	Clinically intact mucosa with bone necrosis only evident in alveolar bone (i.e., only identifiable radiographically)
Stage I	Clinically detectable ORN (exposed bone) which may or may not have radiographic evidence of bone necrosis
Stage II	Radiographic changes extending beyond alveolar bone, may have associated clinical features
Stage III	Advanced ORN with features such as pathological fractures or fistulas identified clinically or radiographically
ORN, osteoradionecrosis	

### Epidemiology

It is difficult to fully assess the number of patients affected by ORN in the UK. Although ORN is not a common complication of radiotherapy, many patients undergo head and neck radiotherapy each year. Between 2018–2020 there were 31,026 new cases of head and neck cancer (HNC) recorded in England; 60% of these patients (18,702) were treated with radiotherapy.^10^

The recorded incidence of ORN within this group varies between different studies. Yang *et al.* reviewed 28 different studies, finding a range of 0–20% ORN incidence.^11^ A systematic review by Nabil *et al.* which included older studies demonstrated an ORN incidence of 7%, while in a cohort of >2,000 patients an incidence of 8% was found.^4^^,^^12^

ORN can severely impact quality of life and has high treatment costs.^5^ HNC patients have significantly increased care costs for at least five years beyond initial curative treatment and the management of ORN can greatly add to these.^13^^,^^14^ Patients with ORN have increased incidence of dysphagia, xerostomia, reduced mouth opening and pain compared to those without.^15^^,^^16^^,^^17^^,^^18^ One study showed 50% of patients with progressive disease rated their quality of life as ‘less than good'.^19^ Figures on mortality are difficult to distinguish given the competing mortality risks among HNC patients, but Chieng *et al.* estimated a 73% survival rate in their cohort of 109 ORN patients at 60 months.^19^

## History and theories of ORN aetiopathogenesis

**Fig. 1:**
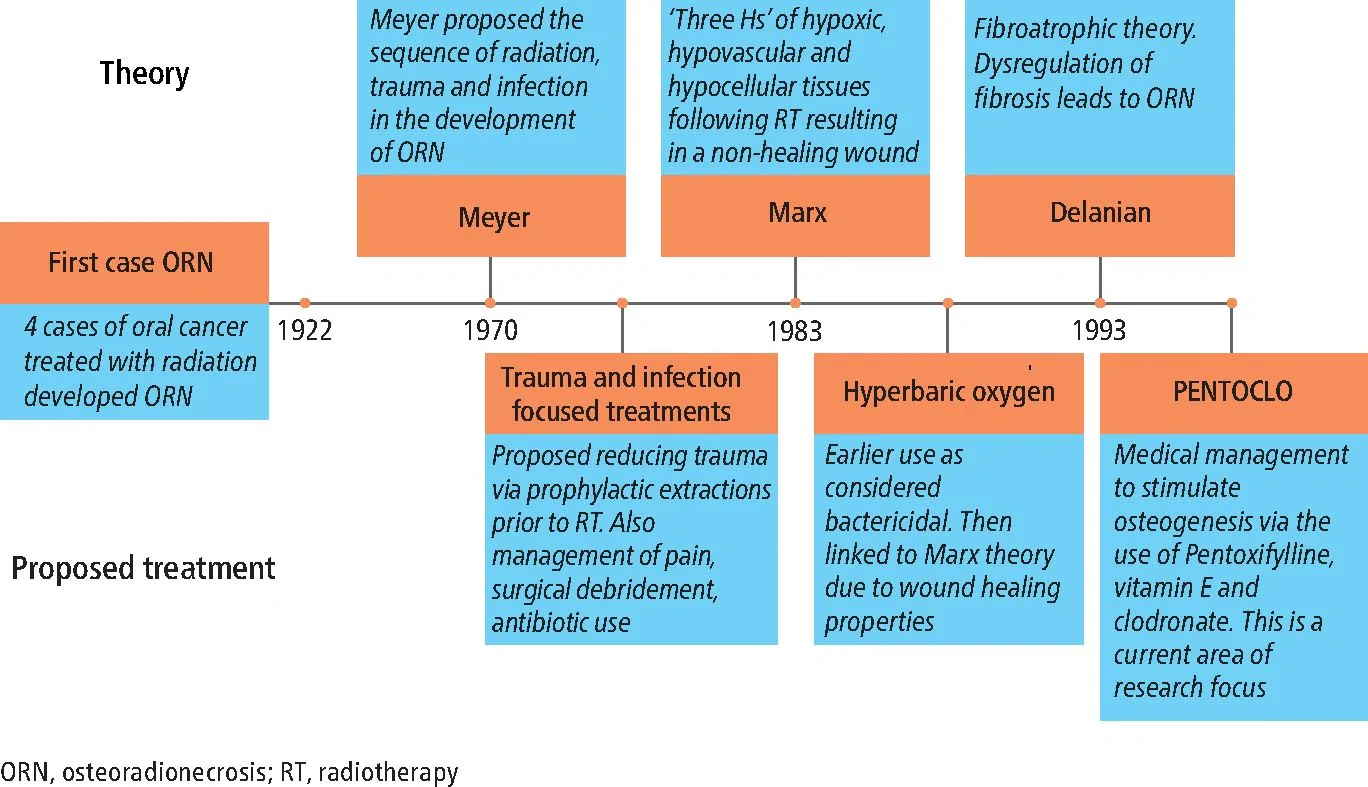
Development of osteoradionecrosis theories and linked treatments over time

ORN was first reported by Regaud in 1922^20^ where he observed radionecrosis in irradiated bone when treating four cases of oral cancer (Fig. 1). ORN occurred spontaneously in three cases and following dental extraction in one. Regaud hypothesised this change was due to bone acting as a ‘secondary radiator' and thereby ‘burning itself' and having resistance to infection, which persists long after radiation treatment.

Subsequently, Meyer described ORN as radiation osteomyelitis with a three-stage process: radiation, then trauma, causing infection.^21^ Vascular changes to bone were also described secondary to radiation. Prevention of post-radiotherapy trauma by extracting teeth before starting radiotherapy, with alveolectomy, was suggested. An association between ORN and radiotherapy dose was also speculated.^22^ Surgical resection was proposed as an early treatment modality along with the early use of flaps to achieve soft tissue coverage.^23^

In the 1980s, Marx proposed a new theory for ORN pathophysiology,^24^ countering the widely held view that ORN was secondary to infection arising following irradiation and trauma. From a case series of 26 patients, nine developed ORN spontaneously without evidence of trauma. His proposed theory of pathogenesis was the ‘Three Hʹ principle, wherein irradiated tissues become hypoxic, hypovascular and hypocellular, resulting in tissue breakdown and a non-healing wound.

Prior to this, hyperbaric oxygen (HBO) was used to treat ORN due to its bacteriostatic properties and promoting vascular proliferation.^25^ Marx is credited with developing an HBO protocol with adjuvant surgical intervention at different stages and an associated ORN classification system. Marx linked his ‘three Hʹ theory to HBO use through improved wound healing.^9^

Since Marx, further understanding of the pathophysiology of ORN has led to the fibroatrophic theory,^26^ which proposes that radiation causes a dysregulated process of fibrosis with three distinct stages. This has led to medical approaches to ORN treatment with ongoing research into the use of pentoxifylline-tocopherol-clodronate combination, a combined therapy consisting of pentoxifylline, vitamin E and clodronate.^27^

## Risk factors

Many risk factors influence whether a patient will develop ORN following head and neck radiotherapy (Table 2).

**Table 2:** Risk factors for ORN

	**Risk factors**			
	**Radiotherapy**	**Tumour**	**Patient**	**Dental**
**Widely accepted**	Dose over 50 Gy	Closer to mandible (i.e., oropharyngeal and oral cavity)	Smoking	Active periodontal disease
	Volume of mandible exposed to radiotherapy	Surgical management of tumour		Hopeless prognosis teeth not removed prior to radiotherapy starting
	Repeat radiotherapy			Dentoalveolar surgery (pre- or post-radiotherapy)
**Emerging/inconsistent evidence**	IMRT vs conventional radiotherapy	HPV positive tumours	Alcohol	Poor oral hygiene
	Number of fractions	Adjunctive chemotherapy	Bisphosphonates (anti-resorption and anti-angiogenic medications)	
			General health: low BMI/diabetes	
BMI, body mass index; HPV, human papillomavirus; IMRT, intensity-modulated radiotherapy				

### Radiation and tumour factors

An increased dose of radiotherapy increases risk of ORN development. Recent ASCO guidelines suggest any patient receiving a dose of head and neck radiotherapy above 50 Gy to maxilla or mandible is at high ORN risk.^5^ Others have suggested thresholds of 54 Gy,^28^ 57.3 Gy,^29^ and 60 Gy.^4^^,^^12^ The relationship between radiotherapy dose and ORN risk has not been fully explored: particularly regarding specific threshold doses.

Reducing ORN rates have partially been attributed to a move from conventional radiotherapy to intensity-modulated radiation therapy (IMRT). Peterson *et al.*^17^ reported ORN rates of 7.4% with conventional radiotherapy and 5.2% with IMRT. Others have corroborated these findings^30^^,^^31^ but not all studies agree.^32^

Reducing the amount of mandible exposed to radiotherapy is also an important consideration as well as the number of fractions radiotherapy is delivered across.^5^^,^^33^ ORN is more likely to develop in the mandible than maxilla, particularly the posterior mandible.^12^^,^^18^ Repeat radiotherapy, rarely carried out due to high complication rates, is associated with increased ORN risk.^34^

The site of cancer is another determinant of ORN risk. Tumours occurring closer to the mandible are more likely to result in ORN likely due to the increased radiotherapy dose received the mandible (e.g., oropharyngeal or oral cavity cancers).^18^ Conversely hypopharyngeal and nasopharyngeal carcinomas have lower ORN rates, despite maxillary and skull base cases of ORN being noted.^11^ Emerging evidence suggests the human papillomavirus status of cancers may also be linked to ORN risk.^35^ This is perhaps related to a younger patient cohort receiving radiotherapy and therefore longer timeframes in which complications may develop.

Furthermore, patients who receive combined modality treatment (surgery and radiotherapy), rather than radiotherapy alone, had a higher risk of developing ORN in several studies.^11^^,^^28^^,^^35^ There is inconsistent evidence whether adjunctive chemotherapy increases ORN risk.^33^^,^^35^^,^^36^

### Patient factors

As smoking and alcohol are key risk factors for oral cancer, there is a higher prevalence of these habits among those exposed to head and neck radiotherapy, which can make exploration of associated risks challenging. Some studies have found no significant link between smoking and alcohol with ORN.^35^^,^^37^ However, multiple studies report higher prevalence of smoking ± alcohol in patients who develop ORN post-radiotherapy.^11^^,^^38^^,^^39^^,^^40^^,^^41^ Overall, smoking (with or without alcohol) is considered a risk factor for ORN development.^4^^,^^5^^,^^41^

A patient's general health is a major risk factor for ORN. Goldwaser *et al.* demonstrated that underweight patients were at higher risk of ORN although there is little recent literature.^37^ Some studies have shown diabetes mellitus to be associated with increased ORN risk.^38^^,^^42^ There is also conflicting evidence surrounding steroid, anticoagulant and bisphosphonate use.^18^^,^^37^^,^^42^^,^^43^

### Dental factors

Oral hygiene, and dental health are further modifiable risk factors associated with ORN.^11^ Patients with active dental disease, particularly periodontitis, have increased ORN risk.^4^^,^^44^ Poorer oral hygiene was seen by Katsura *et al.* in patients who developed ORN compared to those that did not.^18^^,^^39^

Failing to have teeth of hopeless prognosis extracted prior to radiotherapy was a significant risk factor in increased risk of tooth failure and subsequent exposed bone following radiotherapy.^44^ Any dentoalveolar surgery after radiotherapy can increase ORN risk.^38^

## Prevention

Management of ORN is highly challenging and can have a severe impact on patients; therefore, prevention is critical. The restorative-surgical dental team has an important role in preventing ORN by addressing any modifiable risk factors.

### Pre-radiotherapy prevention

A pre-radiotherapy dental assessment is a key part of ORN prevention. Multiple guidelines recommend that this should be an integral part of the oncology pre-treatment assessment, with a restorative dental specialist involved.^5^^,^^45^ This assessment extends beyond simply deciding whether dental extractions are required and should consider the risks of radiotherapy to oral health in a patient-specific manner. It is helpful for the assessing dentist to have information about the planned treatment during this assessment.^5^

Dental assessments should be coupled with prevention and smoking cessation advice. Fluoride delivery should be considered to reduce the risk of radiation caries.^5^^,^^46^^,^^47^ Improvements in pre-radiotherapy oral hygiene and periodontal health are desirable and may reduce the need for post-radiotherapy extractions.^48^

Extraction of poor prognosis teeth should be considered before radiotherapy following careful treatment planning. The importance of reducing ORN risk must be balanced with quality of life and the impact of potentially aggressive dental treatment.^49^ ORN can occur in pre-radiotherapy extraction sites if radiotherapy starts before the socket is fully healed, increasing the importance of long-term dental follow-up.^33^^,^^38^^,^^49^^,^^50^

Studies of US and UK/Irish professionals have helped develop consensus statements to guide treatment planning.^51^^,^^52^ Contemporary treatment planning focuses on tooth retention, and optimisation of tooth prognosis. Extractions frequently involve only teeth of hopeless prognosis if the patient is willing and able to care for their mouth. For example, a tooth with 50% bone loss and furcation involvement may have a 67% ten-year prognosis if appropriately managed.^53^

However, some teeth may need removing. For example, Moore *et al.* suggested that periodontally involved teeth that were grade III mobile should be extracted alongside grade II mobile teeth but only if exposed to >50 Gy.^52^ Other considerations include retained roots, apical pathology, teeth exposed to >50 Gy and if teeth are deemed strategic.^51^

Importantly, due to difficulties in predicting prognosis of teeth, there are no specific guidelines or high-quality evidence regarding which teeth should be extracted prior to radiotherapy. This decision needs to be part of a thorough dental assessment where patient factors are accounted for, and potential risks presented to the patient. Shared decision making is a key aspect of informed consent with emphasis placed on discussing treatment in a patient-centred way following the Montgomery ruling.^54^

Extractions should happen as soon as possible after a decision for radiotherapy is made to allow for increased healing time. Extractions should take place ≥10–14 days before radiotherapy starts, but radiotherapy should not be delayed due to dental treatment needs if it will impact oncology care.^5^^,^^45^^,^^51^^,^^52^ Shorter healing times are associated with an increased risk of exposed bone.^44^

### Post-radiotherapy prevention

Post-radiotherapy treatment should be completed with knowledge of the oncology treatment undertaken and radiotherapy fields and doses. These patients are at high risk for dental disease so prevention regimes should reflect this. Oral hygiene instruction, fluoride, reduced recall interval and smoking cessation are key in the post-radiotherapy phase.^51^

Extractions should be avoided where possible, with endodontic or other restorative options explored, even in cases where ordinarily endodontic treatment would not be considered.^45^ This is mainly based on expert opinion with limited higher-quality evidence available. Dental implants are not usually indicated unless other treatment options have been exhausted.^5^

Despite being recommended by ASCO guidelines, there is limited evidence for use of prophylactic antibiotics to prevent ORN if extractions are required following radiotherapy.^5^ Many of the studies investigating antibiotic use do not have comparator groups. Therefore, no definitive conclusions about efficacy can be made.^55^^,^^56^ Despite this, antibiotics have been recommended both pre- and post-operatively if a tooth has been exposed to high-dose radiation.^5^^,^^45^ Similarly, little evidence exists for using antiseptic rinses.^5^^,^^55^

HBO has been used both for treatment of ORN and prophylactically with post-radiotherapy extractions, with many centres still using it. ASCO recommends HBO is not routinely used prophylactically.^5^ A recent Cochrane review of HBO use was unable to pool data from the three studies included on wound healing after dental extraction or implant placement so no conclusions could be reached.^57^ The HOPON trial was a randomised controlled trial (RCT) comparing HBO use in preventing ORN for patients requiring post-radiotherapy extractions or implant placement. It showed no significant difference in rates of ORN between the HBO and comparator groups.^58^

There is insufficient evidence currently to recommend routine use of blood-derived products such as platelet-rich plasma or fibrin to reduce ORN following post-radiotherapy extractions.^5^^,^^48^^,^^59^ An RCT showed improved mucosal healing post-extraction using photobiomodulation therapy but had insufficient follow-up to assess impact on ORN rates.^5^^,^^60^

The medical prevention of ORN using pentoxifylline and tocopherol (PENTO) before and after post-radiotherapy extraction is an area of emerging research. PENTO is now recommended by ASCO.^5^ Patients must be cancer-free and without certain comorbidities. A systematic review and meta-analysis found ORN rates in patients treated with PENTO was 3.4% compared to 17.6% in controls. However, it was difficult to statistically evaluate these differences due to variation in studies, mostly retrospective cohorts with no comparator group.^61^^,^^62^ Samani *et al.* included a comparator group and found reduced ORN incidence in patients treated with PENTO.^63^

## Case study

A patient (48-year-old man) was assessed prior to oncology treatment by the restorative team. He had an oncology diagnosis of T3N2M0 P16+ squamous cell carcinoma of the left tonsil. The patient was otherwise medically well and had quit smoking over 20 years previously. The restorative team recommended extraction of 46 and a partially erupted impacted 38 which were completed six days prior to starting radiotherapy. His oncology treatment involved 70 Gy of radiotherapy over 35 fractions with three cycles of chemotherapy.

**Fig. 2:**
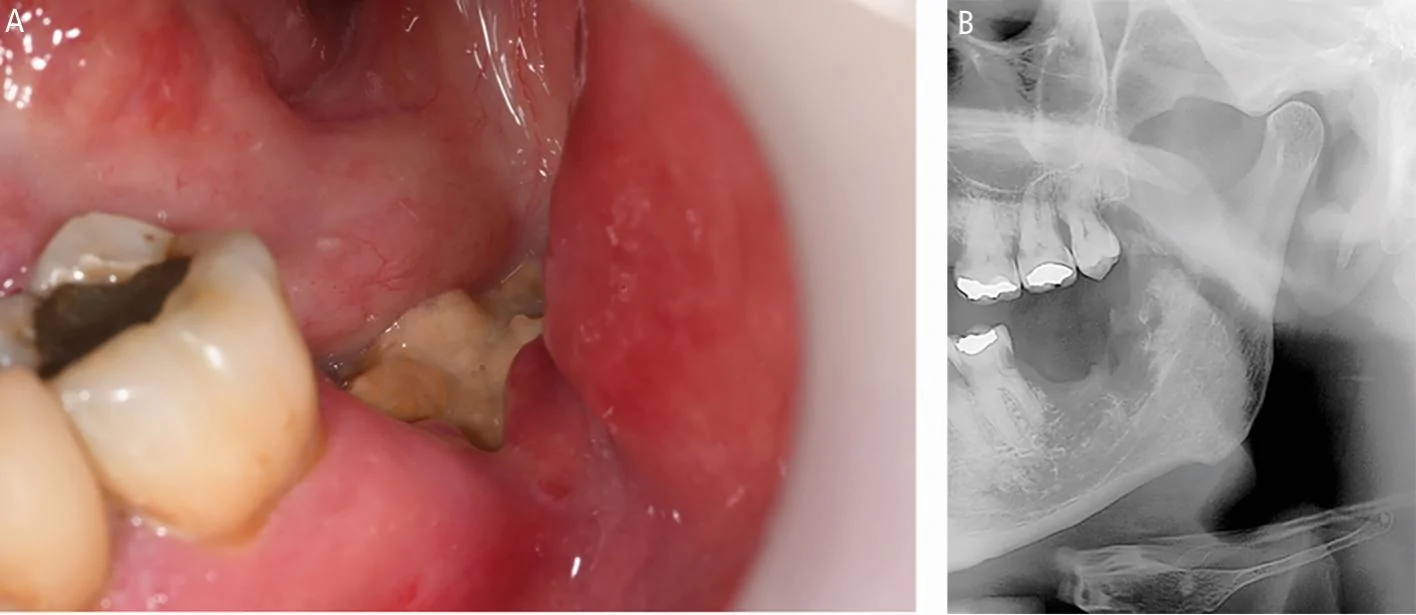
Osteoradionecrosis diagnosed in the left body of the mandible. Consistent with the site of pre-radiotherapy extraction of 38. (A) Clinical photograph of area. (B) Sectional panoramic radiograph

Following completion of chemoradiotherapy the patient was diagnosed with osteoradionecrosis in the left body of mandible, in 38 extraction site five months after radiotherapy completion (Fig. 2).

**Fig. 3:**
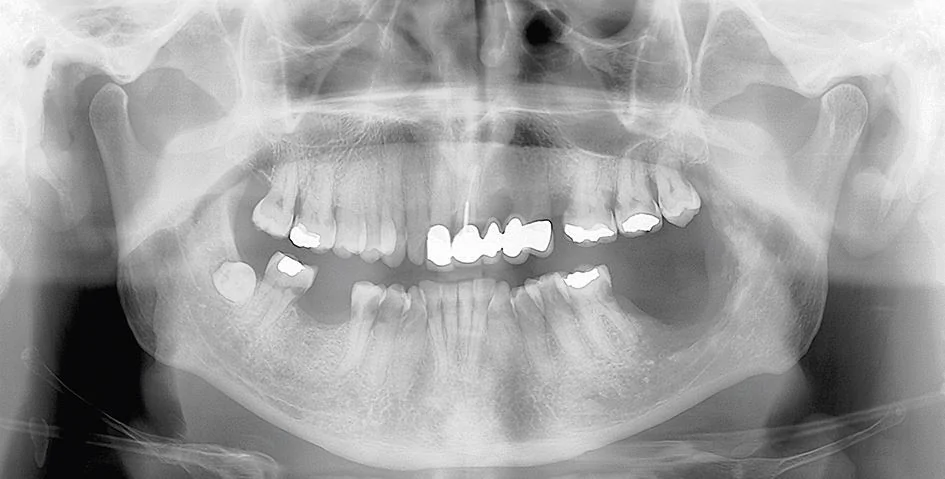
Orthopantomogram showing healing of area of osteoradionecrosis following completion of debridement under general anaesthetic

Treatment of ORN included antibiotic therapy for acute superinfections, debridement under local anaesthetic and further debridement under general anaesthetic seven months after diagnosis (Fig. 3). This stabilised the lesion which has healed with full mucosal coverage and no further bony progression.

**Fig. 4:**
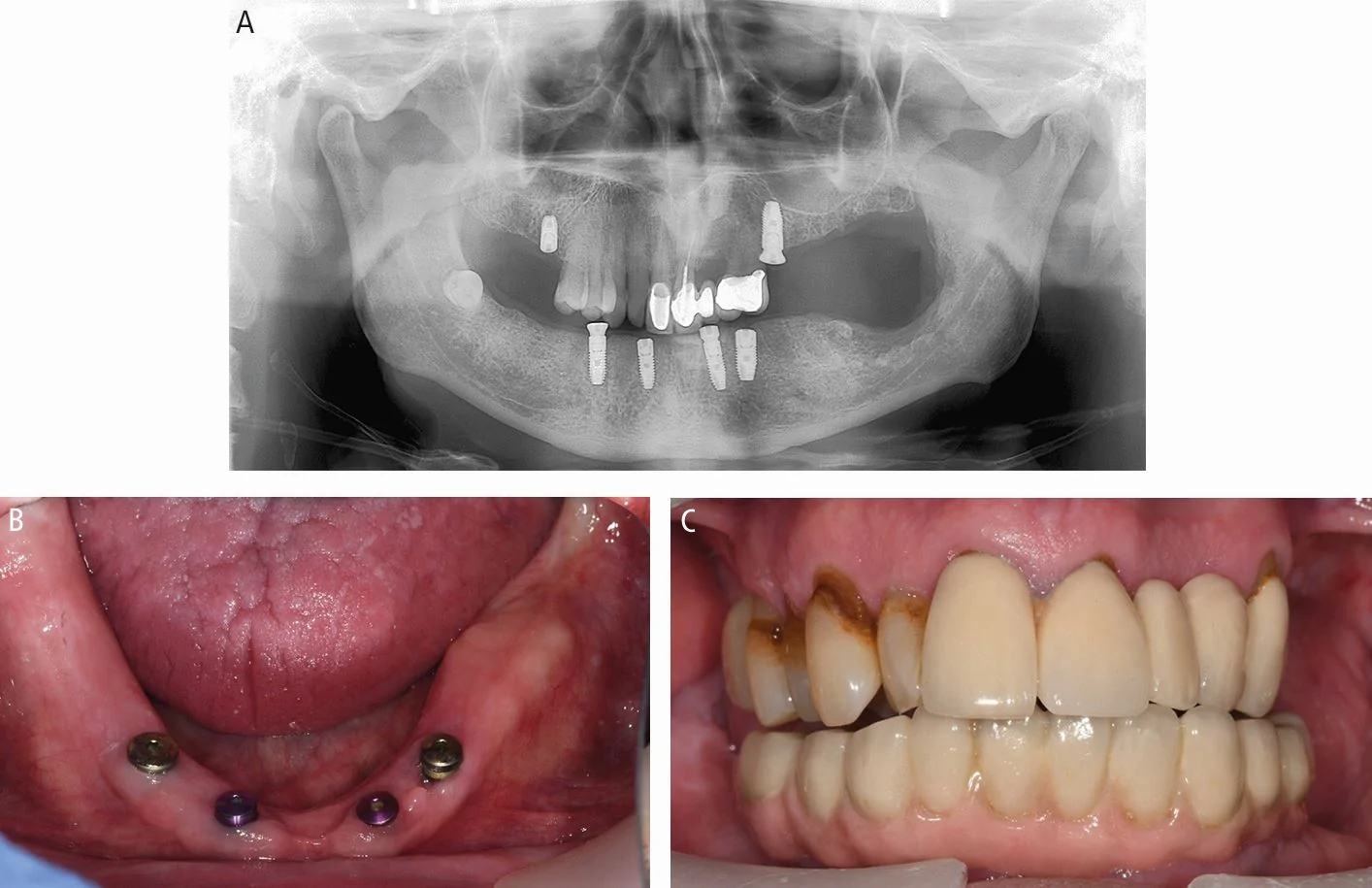
Successful rehabilitation of area with implant-retained fixed bridges. (A) Panoramic radiograph of osteoradionecrosis lesion healing three years post debridement and following implant placement. (B) Clinical photograph of mandibular implants with defect from healed ORN lesion. (C) Clinical photograph of implant retained fixed bridges *in situ*

Restorative treatment initially included management of radiation caries with restorations and endodontic treatment, alongside removable dentures. However, extensive radiation caries, requiring a full lower clearance, and defects from the ORN lesion impacting denture wearing, resulted in a revised treatment plan. Dental rehabilitation included placement of seven implants to ensure a functional occlusion (Fig. 4). The ORN risk assessment prior to implant therapy is outlined below and the patient has been successfully monitored for four years since placement:

- Radiotherapy fields were reviewed to identify areas receiving a radiation dose <50 Gy
- A staged approach allowed time for bony healing of the extraction sockets and ensured implants were placed in bone where previous uncomplicated healing following radiotherapy had occurred.

## General dental practitioner role

General dental practitioners (GDPs) have a key role in the management of patients before and after radiotherapy, with the importance of GDPs in the shared care of these oncology patients well established (Table 3).^45^^,^^64^^,^^65^^,^^66^ Therefore, an awareness of the possible complications head and neck radiotherapy patients may experience is useful. Examples include:

**Table 3:** Prevention of ORN in general dental practice

**Key areas GDPs can aid in prevention of ORN**	
Increased recall frequency based in individual risk assessment	Restoration of radiation caries as required (composite or GIC)
Oral hygiene instruction, tailored to individual patient	Fluoride application including: topical fluoride varnish, prescription of high fluoride toothpaste, fluoride mouthwash use and construction of fluoride trays
Awareness of other complications of radiotherapy e.g., mucositis, trismus, dysphagia and xerostomia	Suggest use of other adjuncts: e.g., GC Tooth Mousse
Diet advice	Full mouth professional mechanical plaque removal at recall intervals when indicated
Surveillance for ORN or cancer recurrence with timely referral to OMFS (consider 2WW pathway)	Referral of patients if post-radiotherapy extractions required or for implant treatment planning
2WW, two-week wait; GDPs, general dental practitioners; GIC, glass ionomer cement; OMFS, oral and maxillofacial surgery; ORN, osteoradionecrosis

- Mucositis
- Radiation caries
- Trismus
- Dysphagia
- Xerostomia
- ORN.^67^

Prevention and surveillance are key roles of GDPs for potential ORN patients. Previous head and neck radiotherapy should be captured in the medical history. Many risk factors for ORN are modifiable. Helping patients to optimise oral hygiene and providing fluoride varnish and high fluoride toothpaste are key preventive therapies.^46^^,^^47^

Treating active dental disease is critical alongside keeping these high-risk patients on shorter recall intervals.^44^^,^^45^^,^^48^^,^^51^ Carrying out routine dental treatment, such as root treatments and prompt management of radiation caries can also reduce the need for post-radiotherapy extractions. Smoking cessation and alcohol reduction advice is also important.^5^^,^^41^

Many post-radiotherapy patients will be under the care of an oral and maxillofacial surgery (OMFS) unit but some may have been discharged to primary care after an appropriate follow-up period. Close surveillance and re-referral (e.g., if a new case of ORN was suspected) is critical. Differentials for ORN include cancer recurrence, osteomyelitis or medication-related osteonecrosis of the jaw.^68^^,^^69^^,^^70^ In these instances, urgent referral may be appropriate, especially if recurrence is suspected.

Advice or treatment from secondary care should be sought if considering extractions. These referrals may be suitable for either oral surgery/OMFS or restorative dentistry, depending on whether restorability assessment of teeth is required. For both extraction and implant cases radiotherapy dose distribution maps are required to assess risk.

## Management of ORN

Management of ORN is usually undertaken within OMFS units. However, an appreciation of management options is helpful when treating these patients in practice.

Conservative management often comprises oral hygiene, analgesia and antibiotics. Long-term doxycycline is sometimes utilised.^71^ Removal of sequestrae or debridement with local anaesthetic is often undertaken.^72^^,^^73^

HBO has long been used for ORN management^9^ but evidence for its use is conflicting, with a recent RCT showing no significant improvement.^74^ Other issues such as expense and accessibility of HBO units mean its use in the UK is decreasing.^75^

Medical management of ORN using pentoxifylline (often in conjunction with tocopherol, antibiotics, clondronate or prednisolone) is also recognised as a non-surgical treatment option. Previous studies have often had no control group, although show some promise.^27^^,^^76^ The RAPTOR trial (randomised controlled trial of pentoxifylline, tocopherol and clodronate in mandibular osteoradionecrosis), an ongoing RCT, is currently looking at this treatment.^77^

Surgical management is typically reserved for complex cases and may involve partial or segmental resection of the affected area often where there are pathological fractures. These resections may leave significant defects requiring reconstruction using reconstruction plates or composite free flaps. These flaps are often taken from the fibula, iliac crest or radial forearm.^5^^,^^73^^,^^78^ Combinations of different treatments can be used to optimise results. However, management is often complex, with surgical management itself a risk factor for further ORN.

## Conclusions

ORN is a complex disease which is the focus of rapidly developing research. Due to difficulties in management of ORN and its significant impact on patients' lives, prevention is critical. There is a clear role for the dentist in shared care for these patients. This includes recognition of potential cases, appropriate referrals to secondary care and preventative measures.

## Data Availability

All data supporting the findings of this study are available within the paper. This work uses data that has been provided by patients and collected by the NHS as part of their care and support. The data is collated, maintained and quality assured by the National Cancer Registration and Analysis Service, which is part of NHS England. The data is taken from the Get Data Out tables'.
